# Factors Associated With the Use of a Salt Substitute in Rural China

**DOI:** 10.1001/jamanetworkopen.2021.37745

**Published:** 2021-12-08

**Authors:** Yishu Liu, Hongling Chu, Ke Peng, Xuejun Yin, Liping Huang, Yangfeng Wu, Sallie-Anne Pearson, Nicole Li, Paul Elliott, Lijing L. Yan, Darwin R. Labarthe, Zhixin Hao, Xiangxian Feng, Jianxin Zhang, Yuhong Zhang, Ruijuan Zhang, Bo Zhou, Zhifang Li, Jixin Sun, Yi Zhao, Yan Yu, Maoyi Tian, Bruce Neal, Hueiming Liu

**Affiliations:** 1 The George Institute for Global Health, University of New South Wales, Sydney, Australia; 2 Research Center of Clinical Epidemiology, Peking University Third Hospital, Beijing, China; 3 National Clinical Research Center for Cardiovascular Diseases, Fuwai Hospital Chinese Academy of Medical Sciences, Shenzhen, Shenzhen, China; 4 The George Institute for Global Health at Peking University Health Science Center, Beijing, China; 5 Peking University Clinical Research Institute, Peking University, Beijing, China; 6 Centre for Big Data Research in Health, University of New South Wales, Sydney, Australia; 7 School of Public Health, Imperial College London, London, United Kingdom; 8 Duke Global Health Institute, and Global Health Research Centre, Duke Kunshan, University, Kunshan, China; 9 Feinberg School of Medicine, Northwestern University, Chicago, Illinois; 10 School of Public Health, Changzhi Medical College, Changzhi, China; 11 Department of Noncommunicable Disease Prevention and Control, Center for Disease Control of Hebei Province, Shijizhuang, China; 12 School of Public Health and Management, Ningxia Medical University, Yinchuan, China; 13 School of Public Health, Xi’an Jiaotong University School of Medicine, Xi’an, China; 14 Department of Evidence-based Medicine, First Hospital of China Medical University, Shenyang, China; 15 School of Public Health, Harbin Medical University, Harbin, China; 16 Sydney Institute for Women, Children and Their Families, Sydney Local Health District, Sydney, Australia

## Abstract

**Importance:**

Lowering sodium intake reduces blood pressure and may reduce the risk of cardiovascular diseases. The use of reduced-sodium salt (a salt substitute) may achieve sodium reduction, but its effectiveness may be associated with the context of its use.

**Objective:**

To identify factors associated with the use of salt substitutes in rural populations in China within the Salt Substitute and Stroke Study, a large-scale cluster randomized trial.

**Design, Setting, and Participants:**

This sequential mixed-methods qualitative evaluation, conducted from July 2 to August 28, 2018, in rural communities across 3 provinces in China, included a quantitative survey, collection of 24-hour urine samples, and face-to-face interviews. A random subsample of trial participants, selected from the 3 provinces, completed the quantitative survey (n = 1170) and provided urine samples (n = 1025). Interview respondents were purposively selected from the intervention group based on their different ranges of urinary sodium excretion levels. Statistical analysis was performed from September 18, 2018, to February 22, 2019.

**Exposures:**

The intervention group of the Salt Substitute and Stroke Study was provided with the free salt substitute while the control group continued to use regular salt.

**Main Outcomes and Measures:**

Knowledge, attitudes, and behaviors regarding the use of the salt substitute were measured using quantitative surveys, and urinary sodium levels were measured using 24-hour urine samples. Contextual factors were explored through semistructured interviews and integrated findings from surveys and interviews.

**Results:**

A total of 1170 individuals participated in the quantitative survey. Among the 1025 participants with successful urine samples, the mean (SD) age was 67.4 (7.5) years, and 502 (49.0%) were female. The estimated salt intake of participants who believed that high salt intake was good for health was higher; however, it was not significantly different (0.84 g/d [95% CI, –0.04 to 1.72 g/d]) from those who believed that high salt intake was bad for health. Thirty individuals participated in the qualitative interviews (18 women [60.0%]; mean [SD] age, 70.3 [6.0] years). Quantitative and qualitative data indicated high acceptability of and adherence to the salt substitute. Contextual factors negatively associated with the use of the salt substitute included a lack of knowledge about the benefits associated with salt reduction and consumption of high-sodium pickled foods. In addition, reduced antihypertensive medication was reported by a few participants using the salt substitute.

**Conclusions and Relevance:**

This study suggests that lack of comprehensive understanding of sodium reduction and salt substitutes and habitual consumption of high-sodium foods (such as pickled foods) were the main barriers to the use of salt substitutes to reduce sodium intake. These factors should be considered in future population-based, sodium-reduction interventions.

## Key Points

**Question:**

What contextual factors may be associated with the use of salt substitutes?

**Findings:**

This mixed-methods qualitative study found high acceptability of and adherence to salt substitutes in rural populations in China. The lack of proper health education, misconceptions about salt, and habitual consumption of high-sodium foods were the main barriers to sodium reduction.

**Meaning:**

This study suggests that, despite the use of salt substitutes, the contextual barriers identified could still hinder sodium reduction and should be targeted in future sodium-reduction strategies.

## Teaser

This qualitative study examined factors associated with the use of a salt substitute rural populations in China within the Salt Substitute and Stroke Study, a large-scale cluster randomized clinical trial.

## Introduction

A careful assessment of contextual factors and human behavior is essential when implementing population health strategies, such as dietary salt reduction to reduce the intake of excessive sodium, which is the top-ranked dietary risk factor associated with cardiovascular diseases.^1,2^ A significant number of strokes are caused by high blood pressure due to overconsumption of sodium.^3,4^ There has been substantial evidence from randomized clinical trials showing that reduced sodium intake leads to a decrease in blood pressure.^5^ However, definitive evidence on whether sodium reduction can reduce incident cardiovascular disease events is lacking, to our knowledge.

To address this need, the Salt Substitute and Stroke Study, a large cluster randomized trial in rural China, is examining the effect of sodium reduction through the use of a salt substitute (75% sodium chloride and 25% potassium chloride) on the risk of strokes. This 5-year study recruited 20 995 participants from 600 rural villages in 5 Chinese provinces.^6^ The salt substitute was dispensed free of charge as a replacement for regular salt, with a sufficient amount provided to the households of the participants in intervention villages. The interim analysis of the trial at the third year of the intervention revealed a significant reduction in systolic blood pressure (–2.65 mm Hg; 95% CI, –4.32 to –0.97 mm Hg; *P* < .001) and an increase in urinary potassium excretion (0.77 g; 95% CI, 0.60-0.93 g; *P* < .001), yet no clear association with urinary sodium excretion (–0.32 g; 95% CI, –0.68 to 0.05 g; *P* = .09).^7^ However, a previous study demonstrated a larger effect of about 7 mm Hg net decrease in systolic blood pressure,^8^ while a meta-analysis on randomized clinical trials of salt substitutes also showed a greater mean reduction in blood pressure as well as clear associations with both sodium and potassium excretion.^9^ We conducted this study to understand the contextual factors and human behaviors associated with the use of salt substitutes and to provide insight into the variation in the trial interim results, with a view to identifying potential barriers to and facilitators of large-scale population use of salt substitutes outside of the trial setting.

## Methods

**Figure 1.**
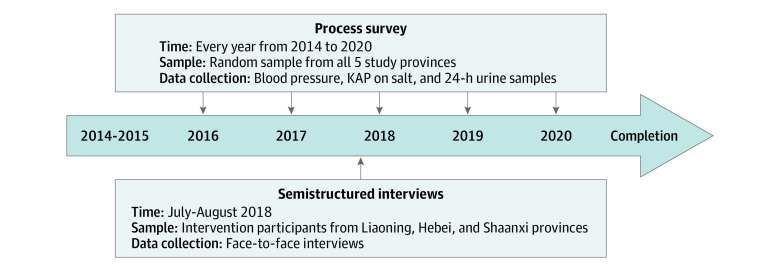
Timeline of the Salt Substitute and Stroke Study and Mixed-Methods Evaluation KAP indicates knowledge, attitude, and practice.

**Figure 2.**
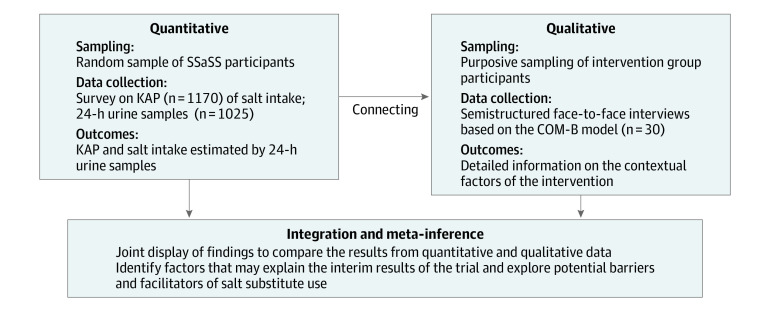
A Sequential Mixed-Methods Study of Salt Substitute Use in the Salt Substitute and Stroke Study (SSaSS) COM-B indicates Capability, Opportunity, Motivation and Behavior; KAP, knowledge, attitude, and practice.

This study was a sequential, mixed-methods evaluation conducted from July 2 to August 28, 2018, at the 3-year follow-up of the Salt Substitute and Stroke Study (ClinicalTrials.gov registration NCT02092090) (Figure 1). A quantitative survey was completed by a subsample of the trial participants randomly recruited across the provinces, with a subsequent qualitative study using semistructured interviews with participants receiving the salt substitute (Figure 2). Participants in the survey provided written informed consent at the beginning of the trial, and participants in the qualitative component of the study were consented separately prior to the interviews. The Salt Substitute and Stroke Study was approved by the institutional review board of Peking University Health Science Center and The University of Sydney Ethics Committee. The qualitative component of this study was approved by the institutional review board of Peking University Health Science Center. We reported the entire mixed-methods study according to the Good Reporting of a Mixed Methods Study (eMethods in the Supplement) and the qualitative part according to the Consolidated Criteria for Reporting Qualitative Research (COREQ) reporting guideline (eMethods in the Supplement).

### Participants of the Quantitative Survey

The main trial was conducted in 5 provinces in northern China, with similar numbers of participants (approximately 4200) from each province (Liaoning, Hebei, Shanxi, Shaanxi, and Ningxia). A process survey, including a structured questionnaire, blood pressure measurements, and collection of 24-hour urine samples, was conducted among a random sample of at least 5% of the participants each year since the start of the trial (see the questionnaire in eMethods in the Supplement). We used the survey conducted in the third year for this evaluation.

### Quantitative Data Collection

Data on knowledge, attitude, and practice (KAP) about salt was captured using an 11-item questionnaire administered by trained interviewers following a standardized protocol. The interviewers were not part of the trial investigator team and were masked to randomized group allocation. Urine samples were collected to measure urinary sodium and potassium excretion; participants were given detailed instructions to collect all voids of urine for the next 24 hours.^7^ Data on demographic characteristics, educational level, and disease history of participants were collected as the baseline data of the trial.

### Quantitative Data Analysis

Statistical analysis was performed from September 18, 2018, to February 22, 2019. The results of the KAP data on salt were summarized as number and percentage, and the amount of salt intake was estimated based on the 24-hour urinary excretion of sodium from each participant and presented as mean (SD) values. The level of salt intake was estimated as follows: salt intake (g/d) = sodium concentration in 24-hour urine samples (mEq/L [to convert to millimoles per liter, multiply by 1.0]) × 24-hour urine volume (L) × 23/1000, accounting for the estimated volumes missed.^10^ Multivariate linear regression was conducted to explore the association between KAP data on salt and salt intake estimated from 24-hour urine samples, adjusting for age, sex, and educational level at baseline. The statistical analysis was performed in STATA, version 14.2 (StataCorp). All *P* values were from 2-sided tests, and results were deemed statistically significant at *P* < .05.

### Participants of the Qualitative Interview

The subsequent qualitative interview was multistaged and purposively sampled to select participants who were in the intervention group. In the first stage, the selection of provinces was based on the mean changes in sodium intake compared with the baseline values estimated from the 24-hour urine samples among participants in each province. Three provinces were selected with a relatively high, medium, and low mean reduction in sodium intake among the participants in those provinces (Hebei, Shaanxi, and Liaoning provinces, respectively). In the second stage, the selection of a village in each province was based on the random selection of the local project coordinators, who, to reduce selection bias, were not aware of the purpose of the interviews. Participants from the selected villages were ranked according to their urinary sodium excretion estimated from 24-hour urine samples. Invitations for interviews were sent out primarily but not only to those in the top and bottom quartiles of urinary sodium excretion, to prioritize the inclusion of individuals with extreme urinary sodium excretion levels. The sampling methods aimed to ensure the inclusion of a broad range of participants with potentially large variations in behaviors and other factors that may be associated with the intervention.

### Qualitative Data Collection

A semistructured interview consisting of 16 questions was conducted face to face with each participant by 2 interviewers (Y.L., Salt Substitute and Stroke Study investigator, and H.C., experienced qualitative researcher independent from the Salt Substitute and Stroke Study). The interview guide is in the eMethods in the Supplement. The sample size of the interview was determined by data saturation. All interviews were audio-recorded after obtaining oral consent from the interviewees. Interviewers visited the kitchens in the participants’ houses and observed their salt and other condiments. Each interview lasted approximately 20 minutes in the participants’ houses with the village physicians interpreting for the interviewers when participants spoke local dialects.

### Qualitative Data Analysis

**Figure 3.**
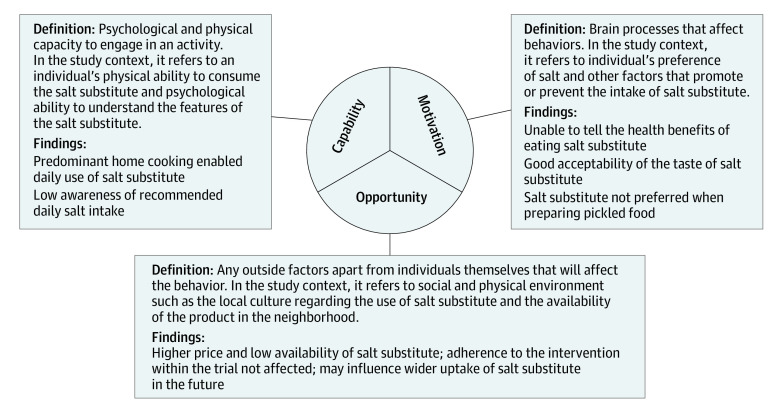
Capability, Opportunity, and Motivation in the Context of the Study

The Capability, Opportunity, and Motivation Behavior (COM-B) model from the Behavior Change Wheel was used as the underlying theory for integration and analysis of findings.^11^ The COM-B model, with its essential constructs of capability, opportunity, and motivation to affect behavior, served as a useful and systematic way of understanding the behavior system (Figure 3). The outer layer of the Behavior Change Wheel would inform how intervention functions and policy categories interact. All the audio recordings of the interviews were transcribed and then deleted. Thematic analysis was used to identify and summarize common themes from the interviews. Two researchers (Y.L. and K.P.) coded the transcripts independently and summarized the main themes under domains of the COM-B model. In the context of this evaluation, *capability* referred to an individual’s physical ability to consume the salt substitute and whether he or she was capable of understanding the knowledge about the salt substitute. *Opportunity* referred to the factors outside the individual’s sphere that would affect the use of the salt substitute. *Motivation* referred to individual’s self-reflected preference of salt and other factors that might provoke the uptake of the salt substitute. Coding results were compared and discussed between the 2 researchers, and a third researcher (H.C.) was involved in the discussion of inconsistency to reach consensus. The coding and analysis were performed using NVivo, version 12 (QSR International).

### Integration of Quantitative and Qualitative Results

Common themes from the qualitative study were triangulated with the quantitative survey data.^12^ Findings were integrated by matching the survey items and interview themes in a joint display for comparisons and meta-inferences.^13^ The coherence of the findings was assessed by confirmation, in which the results from both sources reinforced each other; expansion, in which divergence existed to address different aspects of the phenomenon; and discordance, in which the findings from the quantitative and qualitative studies contradicted each other.^13^

## Results

### Quantitative Results

A total of 1170 participants completed the questionnaire, among whom 1025 had 24-hour urine samples collected (502 [49.0%] women; mean [SD] age, 67.4 [7.5] years; 183 in Liaoning, 222 in Shanxi, 190 in Hebei, 208 in Ningxia, and 222 in Shaanxi) (eTable 1 in the Supplement). Most participants (n = 935 [91.2%]) had hypertension, and 823 (80.3%) were taking antihypertensive agents at baseline. The mean (SD) salt intake estimated by the 24-hour urine samples was 9.6 (5.6) g/d. The understanding of salt and its association with health outcomes varied, and some KAP indicators were weakly associated with salt intake measured from the urine samples (Table 1). Participants who believed that high salt intake was good for health had insignificantly higher estimated salt intake (0.84 g/d [95% CI, –0.04 to 1.72 g/d]) than those who believed that high salt intake was bad for health. The estimated salt intake was 0.87 g/d (95% CI, –1.69 to –0.60 g/d) lower among participants who reported that they tried to reduce their salt intake compared with those who did not.

**Table 1.** Mean Salt Intake of Quantitative Survey Participants and Its Association With Knowledge, Attitude, and Practice^a^

Characteristic	Salt intake, mean (SD), g/d			Mean difference in salt intake vs reference group^b^ (95% CI)			
	Yes	No	Do not know	Yes	*P* value	Do not know	*P* value
Salt good for health	10.5 (7.2)	9.4 (5.0)	9.3 (5.8)	0.84 (–0.04 to 1.72)	.06^c^	–0.12 (–1.08 to –0.04)	.81
No. (%)	193 (18.8)	671 (65.5)	161 (15.7)	NA	NA	NA	NA
Salt intake associated with blood pressure	9.6 (5.1)	9.5 (6.1)	9.6 (6.4)	0.05 (–1.01 to 1.12)	.92	0.15 (–1.02 to 1.33)	.80
No. (%)	631 (61.6)	123 (12.0)	271 (26.4)	NA	NA	NA	NA
Salt intake associated with stroke	10.0 (5.6)	9.8 (6.1)	9.0 (5.4)	0.11 (–0.90 to 1.12)	.84	–0.68 (–1.74 to 0.37)	.21
No. (%)	513 (50.1)	148 (14.5)	363 (35.5)^d^	NA	NA	NA	NA
Try to reduce salt intake	9.4 (5.6)	10.3 (5.7)	NA	–0.87 (–1.69 to –0.60)	.04	NA	NA
No. (%)	794 (77.5)	231 (22.5)	NA	NA	NA	NA	NA
Often eat pickled food	9.7 (5.4)	9.6 (5.7)	NA	–0.21 (–1.07 to 0.66)	.64	NA	NA
No. (%)	198 (19.3)	827 (80.7)	NA	NA	NA	NA	NA
Add extra salt on the table	10.9 (6.4)	9.5 (5.5)	NA	1.36 (0.26 to 2.46)	.02	NA	NA
No. (%)	109 (10.6)	916 (89.4)	NA	NA	NA	NA	NA
Use MSG	9.4 (5.3)	9.7 (5.8)	NA	–0.56 (–1.26 to 0.15)	.12	NA	NA
No. (%)	374 (36.5)	651 (63.5)	NA	NA	NA	NA	NA

Abbreviations: MSG, monosodium glutamate; NA, not applicable.

^a^ A total of 1025 participants with successful collection of 24-hour urine samples are included in the quantitative survey.

^b^ The reference group answered “no” to the question.

^c^ For the multivariate linear regression adjusting for age, sex, and educational level.

^d^ With 1 missing value.

### Qualitative Results

A total of 30 participants (10 from each selected province; 18 women [60.0%]; mean [SD] age, 70.3 [6.0] years) were interviewed to reach data saturation. The highest educational level of the respondents was secondary school (6 [20.0%]), and 24 (80.0%) received a primary school education or lower (eTable 1 in the Supplement). Most respondents (n = 28 [93.3%]) received a diagnosis of hypertension at baseline, with 18 (60.0%) taking antihypertensive medication. The demographic characteristics of participants in both the survey and interview were generally similar. All of the participants were speaking local dialects, and the village physicians were present at all of the interviews to help interpret. Seven common themes were identified from the interviews and were categorized as either barriers or facilitators within the domains of the COM-B model: predominantly home cooking, acceptable taste of salt substitute, lack of understanding about salt substitute, consumption of pickled foods made from regular salt, nonencouraging social environment for salt substitute promotion, low availability of salt substitute or available salt substitute not readily accessible, and sensitivity to higher prices of salt substitutes (eTable 2 in the Supplement).

#### Capability

In the quantitative survey, 794 participants (77.5%) reported that they reduced salt intake in their daily life. Their behaviors matched the analysis of their 24-hour urine samples, which showed that people who self-reported salt reduction had lower salt intake than those who did not. There was a greater percentage (426 of 520 [81.9%]) of people in the intervention group reporting that they reduced salt intake (Table 2). The findings from the interview provided greater insights into the participants’ capability to use the salt substitute. All the interviewed families used the salt substitute in their daily cooking. They cooked most of their meals at home and ate at restaurants only on very rare occasions. The use of the salt substitute was facilitated by the trial participants who were older and living in rural areas predominantly cooking at home. However, only 17.4% of the quantitative survey participants (178 of 1025) and 6.7% of the interview respondents (2 of 30) reported an awareness of the recommended daily salt intake (<6 g/d, according to the Chinese guideline^14^ at the time of the survey).

**Table 2.** Joint Display of Quantitative and Qualitative Findings by COM-B Model Domains^a^

**COM-B domain**	**Quantitative findings, No. (%)**	**Qualitative findings**	**Meta-inferences**
Capability			
Try to reduce salt intake	426 of 520 (81.9)	Predominant home cooking enabled the use of salt substitute on a daily basis.	Expansion: Both demonstration of reduced salt intake and predominant home cooking to use salt substitute confirmed the participants’ capability of salt reduction. However, the lack of knowledge of the recommended amount of salt intake may be a barrier to sodium reduction.
Know that recommended daily salt intake is <6 g	102 of 520 (19.6)	Lack of awareness of recommended daily salt intake	
Opportunity			
Have heard of low-sodium salt	498 of 520 (95.8)	(1) Low availability and available salt substitute not readily accessible; salt substitute cannot be found in village grocery stores. (2) Sensitive to higher prices of salt substitute; prefer the regular salt owing to lower price than the salt substitute	Confirmation: Price sensitivity enhanced the adherence to salt substitute within the trial. The awareness of salt substitute was high in the intervention group as expected. Low availability and price sensitivity may hinder the promotion of salt substitute beyond the trial.
Motivation			
Know that a high salt intake is bad for health	355 of 520 (68.3)	Acceptable taste of salt substitute; the taste, although slightly bitter, was acceptable; some did not notice the bitter taste	Discordance: Quantitative survey data showed relatively good understanding of salt intake and its association with health outcomes. However, qualitative data identified lack of understanding of salt substitute by most respondents. Qualitative data further revealed 2 other factors associated with the use of salt substitute.
Know that the amount of salt intake is associated with blood pressure	332 of 520 (63.9)	Lack of understanding about salt substitute; most respondents cannot tell the potential health benefits associated with using salt substitute	
Know that the amount of salt intake is associated with the risk of stroke	272 of 520 (52.3%)	Salt substitute not preferred when making pickled food because of its bitter taste	
Behaviors			
Often eat pickled food	99 of 520 (19.0)	Regular consumption of pickled food; eating pickled food was very popular in the local dietary habits	Discordance: the rate of pickled food consumption reported in the survey data was much lower than the qualitative data showing that eating picked food was very common. Common use of MSG was identified from both sources.
Add extra salt on the table	55 of 520 (10.6)		
Use MSG	177 of 520 (34.0)	Popular use of MSG; MSG found in most household kitchens	

Abbreviations: COM-B, Capability, Opportunity, Motivation and Behavior; MSG, monosodium glutamate.

^a^ Quantitative and qualitative results from the participants in the intervention group with successful urine collection only. The knowledge, attitude, and practice of the participants in the control group are not presented. Meta-inference includes comparison of quantitative and qualitative findings. Confirmation means findings are consistent. Discordance means findings from quantitative and qualitative data disagree with or contradict each other. Expansion means findings from both sources supplement each other.

#### Opportunity

Good adherence to the intervention (defined as using the salt substitute in all cooking every day) was reported by all the interview respondents. This was matched with a significant increase in urinary potassium excretion. A facilitator was that the salt substitute was provided for free to the intervention group: “I used this salt substitute only. It is free and I do not need to buy salt (regular salt) by myself.”

Despite most of the interview respondents expressing a willingness to buy the salt substitute owing to its potential health benefits, several respondents noted that, in the market, the salt substitute was approximately 20% to 50% higher in price compared with regular salt, and that they would be hesitant about buying this more expensive salt substitute after the completion of this trial: “Salt substitute is much more expensive than the regular salt, I would not buy it.” “I may still buy it if it is good for health. It is more expensive. But how much does it cost to buy this salt all year round?”

#### Motivation

There was a common perception among the interview respondents that the use of the salt substitute was “good for health”: “Yes, I heard that it (salt substitute) is good for health. Then it is probably good and it is free. So we use it all year round.”

However, most of those interviewed failed to articulate the potential health benefits associated with consuming the salt substitute. This diverged from the KAP survey, in which 68.3% (355 of 520) reported that a high intake of salt was bad for health, 63.9% (332 of 520) knew that high salt intake would increase blood pressure, and 52.3% (272 of 520) knew the amount of salt intake was associated with a risk of strokes (Table 2).

In addition, an exaggeration of the health benefits associated with consumption of the salt substitute was common among the interviewees. The salt substitute was believed to prevent colds and improve sleeping disorders and musculoskeletal health, such as relief of back and joint pain: “My back, leg, and arm pain disappeared after using this salt for several years. And I did not get [a] cold in recent years.”

“I had no deep understanding of the salt substitute. My feeling was that I had never had a cold since I ate the salt substitute.”

“I felt good when eating this salt substitute. I reduced 1 tablet of my antihypertensive drug after I had this salt. I think the effect of the salt (salt substitute) is very good.”

The misconceptions of the salt substitute being equivalent to daily prescribed medication (in particular, to the blood pressure–lowering drugs) was common. Even though the salt substitute had a subtly bitter taste (because of the potassium chloride) and reduced saltiness, many of the interviewees expressed a willingness to consume it, suggesting that the taste was highly acceptable. Half of the interview respondents reported no difference in tastes between the salt substitute and the regular salt, whereas the other interview respondents noticed a subtle, lower level of saltiness with the salt substitute. Most interviewees reported that the slight difference in taste was not a major barrier in using the salt substitute in daily cooking. However, interviewees reported not wanting to use the salt substitute in preparing pickled food because of its lack of saltiness. Therefore, regular salt was still commonly used for pickling foods, which were often consumed in high amounts on a daily basis.

#### Behaviors

Pickled foods were the major source of sodium intake other than salt added in cooking (eTable 3 in the Supplement), and consumption of pickled foods was common among interview respondents. However, the quantitative survey and qualitative interview findings diverged regarding the consumption of pickled foods. The reported consumption of pickled foods by the quantitative survey participants was only 19.0% (99 of 520).

In addition, an unintended behavior was the phenomenon of reducing the use of antihypertensive medications without a physician’s advice, as reported by a few interview respondents. These respondents stated that a relief of symptoms due to hypertension, such as dizziness, seemed to be associated with their use of the salt substitute. They therefore decided to discontinue the use of the antihypertensive medications.

## Discussion

Overall, our study provides some insights into the variations in the effect of the salt substitute in the interim results of the randomized clinical trial; although there was a high urinary potassium level indicating adherence to the use of the salt substitute, there was a limited decrease in urinary sodium intake (if the use of regular salt was totally substituted) and a small reduction in blood pressure. We found that contextual factors, including the predominance of home cooking, the acceptable taste of the salt substitute, a limited knowledge about the mechanisms of the salt substitute, the common consumption of pickled foods, and reduced antihypertensive medication use, could potentially explain the variations in the effect of the salt substitute. In addition, the results of this study provide an evidence base for the future design and implementation of salt substitute interventions.

The high urinary potassium level is likely due to the high level of use of the salt substitute, facilitated by the fact that populations living in rural communities in China predominantly cooked at home, that the taste of the salt substitute was highly acceptable, and that the salt substitute was provided for free. In Asian countries such as China, the major dietary source of sodium is discretionary salt use.^15,16^ There are regional variations in the sources of dietary sodium between urban and rural areas in China. Discretionary salt use is higher among people living in rural areas.^17^ Therefore, the predominance of home cooking ensures that replacing the regular salt added to home cooking with the salt substitute is an effective way to reduce sodium intake. Our findings on the taste of the salt substitute being acceptable are consistent with findings from another study testing the acceptability of salt substitutes in northern China.^18^ However, the reduced saltiness of the salt substitute has been recognized as a barrier owing to the habitual preference for salty foods.^19,20^ This preference can be addressed by a gradual reduction of sodium in salt substitutes to achieve an unnoticeable difference in taste over time.^21,22^ Interviewees reported sensitivity to the price of the salt substitute. Adherence to the use of the salt substitute was high because it was supplied free of charge. Price sensitivity has been recognized as a potential barrier in other studies in which the salt substitute was not provided for free.^23,24^ Larger effect sizes of trials providing a free salt substitute were noted^25,26,27,28,29^ compared with a study in which participants were educated to buy and use salt substitutes.^23^ Price sensitivity may hinder the wider uptake of the salt substitute beyond the completion of the trial.

A lack of sufficient knowledge about salt reduction and habitual consumption of pickled foods were the main barriers to sodium reduction, and they can potentially explain the insignificant reduction in urinary sodium in the interim analysis. An evaluation of the national salt reduction strategy in Samoa revealed a lack of capability and motivation among participants to reduce salt intake and to identify foods with a high salt content despite the promotion of salt reduction strategies in health promotion campaigns.^30^ Likewise, a lack of awareness about the recommended amount of salt intake and a misconception of the health benefits of the salt substitute may be associated with higher consumption of the salt substitute than would be normal, to achieve “extra” health benefits. Older people living in rural areas of China generally have low education levels, and greater effort is required to deliver health education on salt reduction in a comprehensive way.

The long-standing tradition of consumption of pickled foods in China contributes significantly to the total amount of sodium intake.^31,32^ The magnitude of sodium reduction achieved by using the salt substitute in home cooking might be attenuated by the common consumption of pickled foods with high sodium content. In addition, the behavior of participants who reduced their use of regularly prescribed antihypertensive drugs without clinical review was not captured by the quantitative survey and may be associated with increases in blood pressure among participants with such behaviors. This behavior may mask the effect of sodium reduction by the use of the salt substitute on blood pressure. Future research on the use of salt substitutes should include this potential behavior for consideration.

### Limitations

There are several limitations of this study. First, the village physicians who delivered the intervention were present at the interviews, which could have had a potential influence on interview respondents to report favorably about the intervention. Second, we designed the qualitative interview and analyzed the results based on the established COM-B model, the hub of the Behavior Change Wheel. The outer layers of the Behavior Change Wheel, including the intervention function and policy categories, were not assessed in this study. Third, the interviews were conducted among participants in the intervention group only. It would be informative to know whether the consumption of pickled foods and the use of antihypertensive medications in the control group were similar to those in the intervention group. The purpose of this study was to evaluate the use of the salt substitute in the intervention group. We do not think the lack of interview data on the control group participants is a major issue.

## Conclusions

This mixed-methods qualitative study provides an in-depth evaluation of contextual factors associated with the use of the salt substitute in a large-scale randomized clinical trial in rural China. Inadequate knowledge of salt reduction and how the salt substitute works to improve health, as well as the habitual consumption of pickled foods, have been recognized as major barriers to decreasing sodium intake to the recommended level. Findings from this evaluation have enabled a greater understanding of the interim trial results and may inform the use of the salt substitute as part of future population-based salt-reduction strategies.

## Notes

Supplement.
eMethods.
**eTable 1.** Characteristics of Qualitative Interview and Quantitative Survey Participants **eTable 2.** Common Themes Identified From the Interviews **eTable 3.** Main Alternative Sources of Sodium Intake by Province From the Interview Respondents
