# Supplementary material for: Factors Associated With the Use of a Salt Substitute in Rural China
Source: JAMA Netw Open. 2021 Dec 8;4(12):e2137745. doi: 10.1001/jamanetworkopen.2021.37745 (PMC8655604; doi:10.1001/jamanetworkopen.2021.37745)
Supplement: Supplement. — eMethods. eTable 1. Characteristics of Qualitative Interview and Quantitative Survey Participants eTable 2. Common Themes Identified From the Interviews eTable 3. Main Alternative Sources of Sodium Intake by Province From the Interview Respondents [file jamanetwopen-e2137745-s001.pdf]

Supplementary Online Content

Liu Y, Chu H, Peng K, et al. Factors associated with the use of a salt substitute in rural China. *JAMA Netw Open.* 2021;4(12):e2137745.
doi:10.1001/jamanetworkopen.2021.37745

eMethods.

eTable 1. Characteristics of Qualitative Interview and Quantitative Survey Participants

eTable 2. Common Themes Identified From the Interviews

eTable 3. Main Alternative Sources of Sodium Intake by Province From the Interview Respondents

This supplementary material has been provided by the authors to give readers additional information about their work.

© 2021 Liu Y et al. *JAMA Network Open.*

eMethods.

Box 1 Good Reporting of A Mixed Methods Study (GRAMMS) checklist (1)

(1)	Describe the justification for using a mixed methods approach to the research question	Page 4-5<br><br>Lines 108-126
(2)	Describe the design in terms of the purpose, priority and sequence of methods	Page 5<br><br>Lines 128-134
(3)	Describe each method in terms of sampling, data collection and analysis	Page 6-9<br><br>Lines 143-223
(4)	Describe where integration has occurred, how it has occurred and who has participated in it	Page 9<br><br>Lines 216-223
(5)	Describe any limitation of one method associated with the present of the other method	Page 16-17<br><br>Lines 385-403
(6)	Describe any insights gained from mixing or integrating methods	Throughout results and discussion<br><br>Table 2

eReference

1. O'cathain A, Murphy E, Nicholl JJJohsr, policy. The quality of mixed methods studies in health services research. 2008;13(2):92-8.

© 2021 Liu Y et al. *JAMA Network Open.*

Consolidated criteria for reporting qualitative studies (COREQ): 32-item checklist (1)

No	Item	Guide questions/description	
**Domain 1: Research team and reflexivity**			
Personal Characteristics			
1.	Interviewer/facilitator	Which author/s conducted the interview or focus group?	Page 8<br>Lines 188-190
2.	Credentials	What were the researcher's credentials? *E.g. PhD, MD*	Page 8, lines 188-190, also available in authors list for authors information
3.	Occupation	What was their occupation at the time of the study?	Page 8, lines 188-190, also available in authors list for authors information
4.	Gender	Was the researcher male or female?	NA, not considered to affect the study
5.	Experience and training	What experience or training did the researcher have?	Page 8, lines 188-190, also available in authors list for authors information
Relationship with participants			

© 2021 Liu Y et al. *JAMA Network Open.*

No	Item	Guide questions/description	
6.	Relationship established	Was a relationship established prior to study commencement?	No. Page 8, lines 194-196, local village doctors who know all the interviewees present at all interviews
7.	Participant knowledge of the interviewer	What did the participants know about the researcher? e.*g. personal goals, reasons for doing the research*	Page 5, lines 135-137, participants know about the goals of the interviews to understand their salt substitute related behaviours and beliefs
8.	Interviewer characteristics	What characteristics were reported about the interviewer/facilitator? e.g. *Bias, assumptions, reasons and interests in the research topic*	Page 8, lines 188-190, lines 192-196
**Domain 2: study design**			
Theoretical framework			
9.	Methodological orientation and Theory	What methodological orientation was stated to underpin the study? *e.g. grounded theory, discourse analysis, ethnography, phenomenology, content analysis*	Page 8-9, lines 198-215, COMB model within the BCW

© 2021 Liu Y et al. *JAMA Network Open.*

No	Item	Guide questions/description	
Participant selection			
10.	Sampling	How were participants selected? *e.g. purposive, convenience, consecutive, snowball*	Page 8, lines 188-196, purposive
11.	Method of approach	How were participants approached? e.*g. face-to-face, telephone, mail, email*	Page 8, lines 188-190, face-to-face
12.	Sample size	How many participants were in the study?	Page 8, lines 191-192
13.	Non-participation	How many people refused to participate or dropped out? Reasons?	NA, none of the participants refused
Setting			
14.	Setting of data collection	Where was the data collected? e.*g. home, clinic, workplace*	Page 8, 193-194, home
15.	Presence of non-participants	Was anyone else present besides the participants and researchers?	Page 8, 194-196, local village doctors
16.	Description of sample	What are the important characteristics of the sample? *e.g. demographic data, date*	Page 10, lines 240-244

© 2021 Liu Y et al. *JAMA Network Open.*

No	Item	Guide questions/description	
Data collection			
17.	Interview guide	Were questions, prompts, guides provided by the authors? Was it pilot tested?	Page 8, 188-190
18.	Repeat interviews	Were repeat interviews carried out? If yes, how many?	No.
19.	Audio/visual recording	Did the research use audio or visual recording to collect the data?	Page 8 lines 192-193, audio recorded and transcribed.
20.	Field notes	Were field notes made during and/or after the interview or focus group?	Yes.
21.	Duration	What was the duration of the interviews or focus group?	Page 8 lines 194-195, about 20 minutes
22.	Data saturation	Was data saturation discussed?	Page 8, lines 192-193
23.	Transcripts returned	Were transcripts returned to participants for comment and/or correction?	No.
**Domain 3: analysis and findings**			

© 2021 Liu Y et al. *JAMA Network Open.*

**No**	**Item**	**Guide questions/description**	
Data analysis			
24.	Number of data coders	How many data coders coded the data?	Page 8 lines 205-207, two coders
25.	Description of the coding tree	Did authors provide a description of the coding tree?	Page 8 lines 198-203
26.	Derivation of themes	Were themes identified in advance or derived from the data?	Page 8 lines 204-205, derived from the data
27.	Software	What software, if applicable, was used to manage the data?	Page 9 lines 215, NVivo
28.	Participant checking	Did participants provide feedback on the findings?	No.
Reporting			
29.	Quotations presented	Were participant quotations presented to illustrate the themes / findings? Was each quotation identified? e.*g. participant number*	Page 11-12 and supplementary materials Table S2
30.	Data and findings consistent	Was there consistency between the data presented and the findings?	Yes. Table 2

© 2021 Liu Y et al. *JAMA Network Open*.

No	Item	Guide questions/description	
31.	Clarity of major themes	Were major themes clearly presented in the findings?	Page 10-13, Table 2 and Table S2
32.	Clarity of minor themes	Is there a description of diverse cases or discussion of minor themes?	Page 13 lines 318-323

eReference

1. Tong A, Sainsbury P, Craig J. Consolidated criteria for reporting qualitative research (COREQ): a 32-item checklist for interviews and focus groups. International Journal for Quality in Health Care. 2007;19(6):349-57.

© 2021 Liu Y et al. *JAMA Network Open.*

KAP questionnaire

SSaSS **Form P** Process Indicator Survey

Centre number Pt number
Registration Number |__|__|__|-|__|__|
Month and year of birth |__|__|__|__| |__|__|
y y y y m m

"Complete EVERY question. Do not leave any question unanswered

1. Clinical assessment

1.1	| | | | | | | | | (yyyy mm dd)	Assessment Date
1.2	| | | |/| | | |	Blood pressure I (systolic/diastolic) mmHg
1.3	| | | |/| | | |	Blood pressure II (systolic/diastolic) mmHg
1.4	|__|__|__|.|__|	Weight (kgs)
1.5	Y N	Selected for urine sample *(If yes, provide materials and instructions)*
1.6	Y N	Completed EQ-5D form

2. Concomitant medications

(Tick " YES" to medications used for most days in the last month)

	yes	no	
2.1	Y	N	Diuretic
2.2	Y	N	Angiotensin converting enzyme inhibitor or angiotensin II receptor antagonist
2.3	Y	N	Alpha-blocker
2.4	Y	N	Beta-blocker
2.5	Y	N	Calcium antagonist
2.6	Y	N	Other antihypertensive agent (including traditional Chinese medicine)
2.7	Y	N	Statin and/or other lipid lowering agent
2.8	Y	N	Aspirin and/or other anti-platelet agent
2.9	Y	N	Oral anticoagulant

3. Knowledge, attitudes and behaviours related to salt

	Yes	No	d/k	
3.11	Y	N	DK	Is a high salt intake good for your health?
3.21	Y	N	DK	Does how much salt you eat affect your blood pressure?
3.31	Y	N	DK	Does how much salt you eat affect your risk of stroke?
3.41	|__|		DK	What is the daily recommended salt intake for adults? <3g, <6g, >6g or >9g?
3.51	Y	N		Do you try to reduce the amount of salt you eat?
3.52	Y	N		Do you eat pickled vegetables most days?
3.53	Y	N		Do you add salt to most meals?
3.54	Y	N		Do you add MSG to most meals?
3.55	Y	N		Do you try to eat less of a food if it is very salty?
3.56	Y	N		Have you heard about low sodium salt?
3.57	→ Y	N		Does your household use low-sodium salt?

© 2021 Liu Y et al. *JAMA Network Open.*

Interview Guide

Province:

County:

Village:

Basic information

Ask about interviewee's demographic information: age, medical history, etc

Eating Habit

1. Do you usually eat at home?

[How many people eat at home? Who cooks?]

2. Other than salt, do you use any other condiment when cooking?

[MSG, soy source, etc]

3. Do you often buy prepared food?

[What types of prepared food? Frequency? Where? Who?]

4. Do you often finish all the dishes including the soup (ort)?

[Who drinks soup?]

5. Do you often eat pickled food? If yes, what types of salt do you use to prepare the pickled food?

[What types? If prepare pickled food at home, how much salt used?]

6. How much salt do you usually use for cooking one dish? Have you changed the amount of salt use (compared to 2 years ago, i.e. before the trial)?

[Use spoons to estimate; ask how long a pack of 200g/400g salt last]

7. How do you feel about the taste of the low sodium salt substitute? If feel less salty, what do you do?

[If add extra salt substitute, if add usual salt or other salty condiment]

8. Do you think you preferred salty taste?

[Self-reflection of the saltiness they prefer]

9. Do you think the amount of salt you eat changes according to seasons?

[Are there any differences between winter and summer?]

10. Does the distributed salt substitute sufficient for your daily use?

[Do you have left over/extra? If yes, do you share with others?]

Attitudes

1. What do you think about the low sodium salt substitute?

© 2021 Liu Y et al. *JAMA Network Open.*

[Do you feel it good? In what way it is good? In terms of health? Disease prevention? Or other perspective? If different from usual salt?]

2. What effect do you think the low sodium salt substitute has?

[If affect blood pressure, risk of stroke? Or other conditions]

3. Do you know any of your neighbour who eat the low sodium salt substitute? Do you know how they think about it?

[Neighbours not participating in the trial]

Environment

1. Can you buy the low sodium salt substitute in the village grocery shop? If yes, how much?

[Supermarkets in the town? How far? Do you often travel to supermarkets further away? Are there price differences between salt substitute and usual salt? Will you buy it if cost more?]

2. Does the village doctor (who distribute the salt substitute) educate you about the salt substitute? Or ask you to eat less of salt?

[If they listen to health advices]

3. Do you have other reflection or points would like to share regarding this study and salt substitute?

[Will you buy salt substitute after the completion of this trial, i.e no longer provided for free]

© 2021 Liu Y et al. *JAMA Network Open.*

eTable 1. Characteristics of Qualitative Interview and Quantitative Survey Participants

**eTable 1: Characteristics of qualitative interview and quantitative survey participants.**		
**Characteristics**	**Quantitative survey N = 1025**	**Qualitative interview N = 30**
Age (mean, SD, years)	67.4 (7.5)	70.3 (6.0)
Gender (n, %)		
Male	523 (51.0%)	12 (40.0 %)
Female	502 (49.0%)	18 (60.0 %)
Literacy (n, %)		
Primary school or lower	750 (73.2%)	24 (80.0 %)
Secondary school	275 (26.8%)	6 (20.0 %)
Disease history at baseline (n, %)		
Hypertension	935 (91.2%)	28 (93.3 %)
Ischaemic heart disease (IHD)	177 (17.3%)	2 (6.7 %)
Diabetes mellitus	109 (10.6%)	4 (13.3 %)
Transient ischaemic attack (TIA)	91 (8.9%)	5 (16.7 %)
Peripheral arterial disease	38 (3.7%)	3 (10.0 %)
Congestive heart failure	18 (1.8%)	1 (3.3 %)
Medication use at baseline (n, %)		
Antihypertensive agent*	823 (80.3%)	18 (60.0 %)
Aspirin and/or other anti-platelet agent	399 (38.9%)	10 (33.3 %)
Statin and/or other lipid lowering agent	122 (11.9%)	1 (3.3 %)
Oral anticoagulant	16 (1.6%)	3 (10.0 %)
Participants (n=1025) with successful collection of 24 hour urine samples are included in the quantitative survey. * antihypertensive agent includes diuretic, angiotensin converting enzyme inhibitor or angiotensin II receptor antagonist, alpha-blocker, beta-blocker, calcium antagonist and other antihypertensive agent (including traditional Chinese medicine).		

© 2021 Liu Y et al. *JAMA Network Open.*

eTable 2. Common Themes Identified From the Interviews

eTable 2: Common themes identified from the interviews.

	Domains in BCW	Common themes	Quotations
Facilitators	Capability	Predominant home cooking	"We always cook and eat at home." --Participant ID 124908, 66 years old, male<br>"For most of times, we cook at home every day. Eating in the restaurants is very rare." --Participant ID 124925, 64 years old, female
	Motivation	Acceptable taste of salt substitute	"I don't taste any difference (of the salt substitute) from the normal salt. They are the same taste to me." --Participant ID 101408, 63 years old, female<br>"It's (salt substitute) slightly less salty compared to the normal salt. But it doesn't matter, its taste is acceptable for me." --Participant ID 155020<br>"It tastes good, the same as the normal salt." --Participant ID 155011, 82 years old, female<br>"I can taste some bitter, but it's ok." --Participant ID 101434, 73 years old, male
Barriers	Motivation	Lack of understanding about salt substitute	"I heard that it is good for health. Then it is probably good and it is free. So we use it all year round." --Participant ID 101411, 64 years old, male<br>"I do not think this salt affect my blood pressure or my health." --Participant ID 124908, 66 years old, male
	Motivation	Consumption of pickled food made from the normal salt	"This salt cannot be used to make pickles, it is not salty enough." --Participant ID 101409, 65 years old, male<br>"It does not go well in pickle making, we use the normal salt instead." --Participant ID 124926, 59 years old, male
	Opportunity	Non-encouraging social environment for salt substitute promotion	"No one in the village except for those participated in the study eat low sodium salt. We never tell others about the supply of salt (salt substitute) because it is free of charge. We are afraid that others may ask for it if we tell them." --Participant ID 124916, 70 years old, female<br>"We do not talk about this salt substitute to our neighbours." --Participant ID 101406, 80 years old, male
	Opportunity	Low availability and available salt substitute not readily accessible	"We do not have salt substitute in the village shops. I heard from someone else that it is available in the supermarkets in the town. I do not travel to the town very often. I can only ask my son or daughter to bring some when they travel to the town. But it is too troublesome, I can buy a pack of normal salt easily from the village shops." --Participant ID 101428, 76 years old, female

© 2021 Liu Y et al. *JAMA Network Open*.

	Opportunity	Price sensitive to higher prices of salt substitute	“Salt substitute is much more expensive than the normal salt, I would not buy it.” --Participant ID 101410, 63 years old, male<br>“It is costly compared to the normal salt. I probably will not buy it if you don’t provide it for free.” --Participant ID 124926, 59 years old, male

© 2021 Liu Y et al. *JAMA Network Open.*

eTable 3. Main Alternative Sources of Sodium Intake by Province From the Interview Respondents

**eTable 3: Main alternative sources of sodium intake by province from the 30 interview responds.**			
	**Mean difference of 24 hour urinary sodium compared to baseline**		
	**Hebei (-0.97g/24 hour)***	**Shaanxi (-0.26 g/24 hour)***	**Liaoning (0.15 g/24 hour)***
	**Percent of interviewees in each province**		
**Pickled vegetables**	60% Use both normal and salt substitute	60% Commonly use normal salt	90% Commonly use normal salt
**Pickled meat**	10%	100% Commonly use salt substitute	0%
**Pickled source**	0%	0%	100% Commonly use normal salt
**Soy source**	80%	70%	100%
**MSG†**	30%	90%	70%

*Mean difference of 24 hour urinary sodium from 3 year process indicator surveys of Hebei (n=190), Shaanxi (n=222) and Liaoning (n=183) provinces compared to their baseline.
†MSG: monosodium glutamate.

© 2021 Liu Y et al. *JAMA Network Open.*
